# Spatiotemporal clustering of highly pathogenic avian influenza (HPAI) H5N1 at the wild waterfowl-poultry interface: Vector-specific spillover risks in the U.S., 2022–2025

**DOI:** 10.1371/journal.pone.0345354

**Published:** 2026-09-23

**Authors:** Csaba Varga

**Affiliations:** 1 Department of Pathobiology, College of Veterinary Medicine, University of Illinois Urbana-Champaign, Urbana, Illinois, United States of America; 2 Department of Biomedical and Diagnostic Sciences, College of Veterinary Medicine, University of Tennessee, Knoxville, Tennessee, United States of America; National Museums of Kenya, KENYA

## Abstract

The emergence of the highly pathogenic avian influenza (HPAI) H5N1 clade 2.3.4.4b in North America, beginning in February 2022, has highlighted the dynamic, unpredictable, and regionally variable risk of infections. Studies are needed to assess the spatiotemporal clustering of HPAI H5 at the interface between wild waterfowl and commercial poultry to better understand and mitigate this risk. Publicly available data on HPAI H5 detections in wild birds and commercial poultry from January 2022 to January 2026 were analyzed at the county level. Retrospective space-time permutation models were used to identify and scan for clusters with higher-than-expected detection rates. A total of 17,091 HPAI H5 detections were reported in wild birds across 1,467 county-level locations. Four species, Mallard (*Anas platyrhynchos*) (2,848 detections, 16.66%), Canada goose (*Branta canadensis*) (1,496, 8.75%), Green-winged teal (*Anas carolinensis*) (1,364, 7.98%), and Snow goose (*Anser caerulescens*) (1,084, 6.34%), accounted for 39.73% of detections. In commercial poultry, 532 outbreaks in turkey operations, 148 outbreaks in table-egg layer operations, 99 outbreaks in broiler chicken operations, and 89 outbreaks in commercial duck operations were reported, respectively. Several spillover events followed an east-to-west expansion. In early 2022, mallard detections preceded outbreaks in Northeast egg-layer and duck farms, while snow goose detections in the Upper Midwest coincided with turkey farm outbreaks. In the Pacific and Mountain West during summer 2022, detections in Canada geese overlapped with turkey farm outbreaks. A resurgence occurred in the Midwest (2025), with snow and Canada goose detections overlapping severe outbreaks in turkey and layer flocks. Additionally, in the Upper Midwest, Canada goose and mallard detections overlapped with outbreaks in commercial duck farms during fall-winter 2025. The study findings demonstrate distinct vector-based transmission dynamics of HPAI H5 at the wild waterfowl-poultry interface. Farm biosecurity strategies must adapt to these recurrent, vector-specific risks.

## Introduction

Avian influenza is an influenza Type A virus, which is classified into different strains based on two viral surface proteins: hemagglutinin (H) and neuraminidase (N). In poultry, highly pathogenic avian influenza (HPAI) viruses are almost always of the H5 or H7 hemagglutinin types, and they can cause severe morbidity and mortality, as well as severe economic concerns [1]. Avian influenza can undergo genetic drift and shift, forming new viral strains, and allowing it to adapt to new host species [2]. Wild waterfowl are considered natural hosts for the virus and can transmit it via migratory flyways, posing a risk to commercial poultry operations [3,4].

The emergence of HPAI strains in North America, specifically the H5N1 clade 2.3.4.4b, beginning in February of 2022, has reinforced how dynamic, unpredictable, and regionally variable the HPAI risk is [5]. Since early 2022, HPAI H5 detections have been confirmed in commercial and backyard poultry flocks, wild waterfowl, wild mammals, and dairy cattle across multiple states in the United States of America (U.S.) [6,7]. Since the start of the HPAI H5 outbreak in commercial and backyard poultry flocks on February 8^th^, 2022, depopulation of over 195 million birds in the U.S. has been reported to date [8].

Transmission of HPAI H5 from wild birds to commercial and backyard poultry can occur through both direct contact and indirect pathways, including contact with infected waterfowl, contaminated environment, fomites, equipment, vehicles, and personnel [9]. These transmission routes contribute to the introduction of the virus into commercial poultry operations and viral transmission within and between farms [10].

Unlike previous HPAI viral strain introductions into the U.S. via migratory flyways, which were restricted to seasonal waves, the current lineage has established endemicity in wild waterfowl populations across the U.S., posing a continuous risk to commercial poultry farms [3]. The current HPAI H5 strains show heterogeneity in vector competence and migratory behavior, are stable in the environment, have expanded their host tropism, including spillover into mammals, and can cause die-offs in waterfowl [11]. Although the risk to the public remains low, sporadic human H5N1 infections have been reported in individuals with direct exposure to infected animals, underscoring the zoonotic potential of these viruses and the need for integrated surveillance across wildlife, livestock, and human populations [12].

The circulation of HPAI viruses in wild waterfowl populations has reinforced the importance of farm biosecurity as a measure for preventing virus introduction into poultry flocks. Because wild waterfowl detections may precede outbreaks in commercial poultry, surveillance of wild waterfowl can provide an early warning for implementing biosecurity measures [13,14]. Consequently, periods of elevated HPAI activity in wild waterfowl should prompt enhanced biosecurity on poultry farms, including the exclusion of wild birds from poultry houses, feed, and water sources; the restriction of farm access; the use of dedicated clothing and footwear; and the routine cleaning and disinfection of vehicles, equipment, and personnel [15].

Current HPAI surveillance systems in the U.S. operate in silos, with data fragmented across monitoring data sets from wild birds [16] and commercial poultry operations [8]. These data silos prevent the generation of cross-sectoral models and real-time risk forecasts that could guide proactive decision-making and early interventions. Moreover, the spatiotemporal overlap among HPAI H5 detections in specific waterfowl species and outbreaks in different commercial poultry sectors is underdefined. Current surveillance often aggregates “wild birds” into a single risk variable, does not account for the distinct ecological roles that each wild waterfowl species plays, nor does it distinguish between the HPAI H5 transmission risk of long-distance migrants versus synanthropic bridge species [17,18].

Despite extensive research on HPAI H5 virus evolution, wild bird surveillance, poultry outbreaks, and the role of migratory flyways in disease dissemination, studies evaluating the spatiotemporal associations between species-specific wild waterfowl HPAI H5 detection clusters and commercial poultry HPAI H5 outbreak clusters at the national scale remain limited. To our knowledge, no previous study has characterized space-time clustering patterns among major wild waterfowl hosts and commercial poultry sectors simultaneously across the contiguous United States. To address this gap, we integrated HPAI H5 surveillance data collected between January 2022 and December 2025 from wild birds and commercial poultry operations to characterize the spatiotemporal interface between four frequently detected waterfowl species, Mallard (*Anas platyrhynchos*), Canada goose (*Branta canadensis*), Snow goose (*Anser caerulescens*), and Green-winged teal (*Anas carolinensis*), and four major commercial poultry sectors: turkey, table egg layer chicken, broiler chicken, and commercial duck. We used disease mapping and retrospective space-time permutation scan statistics to identify significant clusters of HPAI H5 detections and outbreaks and to evaluate spatiotemporal associations between wild waterfowl and commercial poultry clusters across major migratory flyways. We hypothesized that both wild waterfowl HPAI H5 detections and poultry outbreaks would exhibit significant space-time clustering, and that clusters occurring within the same migratory flyways would demonstrate spatial and temporal associations indicative of potential epidemiological connectivity. Furthermore, we expected these associations to differ among waterfowl species and poultry production sectors. The goal of this study was to generate information that can support animal health stakeholders in developing surveillance strategies, early warning systems, and location- and species-specific biosecurity measures to reduce the impact of HPAI H5 on U.S. poultry production systems.

## Materials and methods

### Data source

Wild bird HPAI surveillance is overseen by the United States Department of Agriculture (USDA), Animal and Plant Health Inspection Service (APHIS), which collects data on laboratory-confirmed HPAI H5 detections submitted by federal, state, tribal, and partner agencies across the United States. The samples are collected nationwide to represent the national status of HPAI H5 and are obtained through the investigation of morbidity and mortality events, surveillance in live wild birds, hunter-harvested birds, use of sentinel species, and environmental sampling [16,19]. In commercial poultry, HPAI is a nationally reportable disease, and each confirmed and suspected case must be reported to APHIS and State animal health officials [20].

Publicly available data on HPAI H5 detections in wild birds [21] and commercial poultry [8], between January 1^st,^ 2022, and January 9^th,^ 2026, for wild birds and February 08, 2022, and January 16^th^, 2026, for commercial poultry, were obtained from the APHIS HPAI detection dashboard. Separate downloadable datasets for wild birds and commercial poultry were retrieved in CSV format and used for subsequent analyses. The data included information on detection date, state, county, species (wild birds), and poultry production type (commercial broiler, layer, turkey, ducks).

Outbreaks were analyzed as discrete events rather than incidence rates because comprehensive national denominator data, including species-specific wild waterfowl populations, poultry populations, and numbers of commercial premises at risk by poultry sector and period, were not available. Accordingly, the retrospective space-time permutation scan statistic was used to identify significant concentrations of outbreak occurrences in space and time without requiring population-at-risk information.

### Statistical analysis

The R software (Version 4.5.2) [22] and the RStudio (Version 2026.01) platform were used for data management and descriptive statistics. Disease maps and spatial statistical analyses were conducted in ArcGIS Pro 10.7.1 (Environmental Systems Research Institute, Inc., Redlands, CA, USA). All maps were generated in ArcGIS Pro 10.7.1 using county-level HPAI surveillance data and U.S. Census Bureau state boundary shapefiles.

The HPAI H5 detections were analyzed at the county level. For each county centroid, latitude and longitude were calculated and represented as point features for spatial analysis. The analysis was restricted to counties within the contiguous U.S. (48 states and the District of Columbia, excluding Alaska and Hawaii) to comply with distance-based analyses. The data were projected to the NAD 1983 (2011) USA Contiguous Albers Equal Area Conic coordinate system to ensure accurate distance calculations.

For each wild waterfowl species and poultry operations, choropleth point maps were constructed using divergent colors and natural breaks (Jenks) classification with 5 categories. Inverse distance weighted interpolation was used to complement choropleth mapping of wild waterfowl HPAI H5 detections and to illustrate the spatial intensity of detections and estimate patterns in areas without reported data.

Retrospective space-time analysis was conducted, scanning for clusters with high rates using the space-time permutation model in the SaTScan software (Version 9.6) [23]. This model does not require population-at-risk data, and it was appropriate for our study because information on the total number of tests performed and the background population was unavailable. A cylindrical scanning window was applied, where the circular base represented the geographic area and the height represented the time interval. The maximum spatial cluster size was set to 50% of the population at risk, the minimum temporal cluster size was defined as 1 month, and the maximum temporal cluster size was set to 50% of the study period. Statistical significance (p ≤ 0.05) was assessed using Monte Carlo hypothesis testing with 999 replications. The observed-to-expected (O/E) ratio was calculated for each space-time cluster to quantify excess risk, with values greater than 1 indicating a higher-than-expected number of detections within the cluster window under the null hypothesis of random space-time distribution. All statistically significant space-time clusters were visualized in maps.

## Results

### Distribution of H5 HPAI detections in wild birds

**Fig 1:**
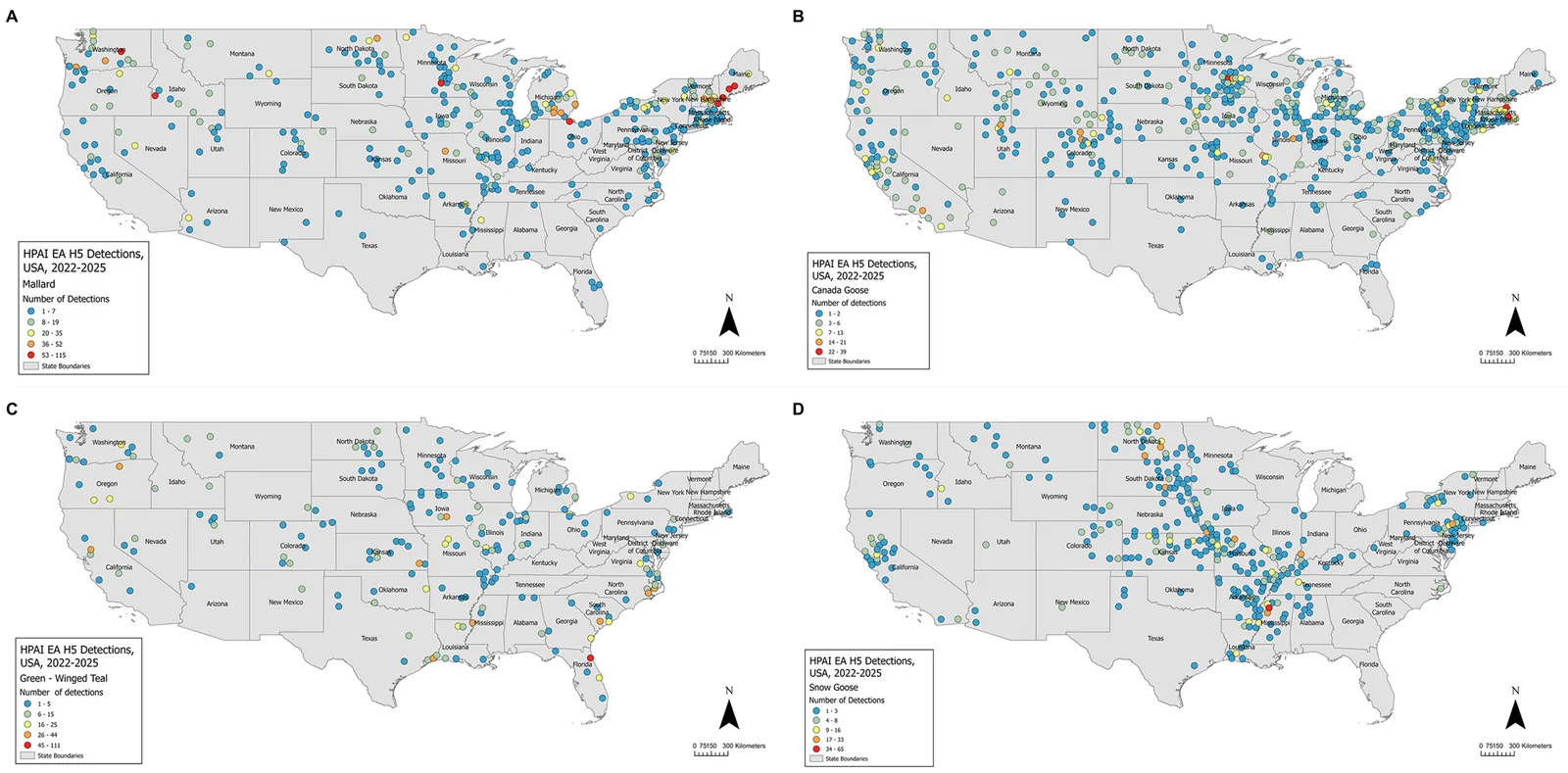
Distribution of highly pathogenic avian influenza (HPAI) H5 detections across the U.S. by wild waterfowl species, 2022-2025. The map highlights detections in four wild waterfowl species: (A) Mallard, (B) Canada goose, (C) Green-winged teal, and (D) Snow Goose. Divergent colors are used, with red indicating high and blue indicating low detection areas.

Between January 1, 2022, and January 09, 2026, 17,091 laboratory-confirmed detections of H5 HPAI were reported in wild birds across 1,467 county-level locations in 48 U.S. contiguous states and the District of Columbia. Four species, Mallard (*Anas platyrhynchos*) (2,848 detections, 16.66%), Canada goose (*Branta canadensis*) (1,496, 8.75%), Green-winged teal (*Anas carolinensis*) (1,364, 7.98%), and Snow goose (*Anser caerulescens*) (1,084, 6.34%), accounted for 39.73% of detections. These four wild waterfowl species were selected for further analysis because they accounted for 39.73% of all laboratory-confirmed detections during the study period and exhibited broad geographic distributions across multiple U.S. migratory flyways. In addition, each species had enough detections to support robust species-specific space-time cluster analyses. The selection was therefore based on data availability, geographic representation, and statistical considerations rather than the assumption that these species were the only hosts involved in HPAI transmission. Other waterfowl species may also contribute to virus maintenance and spread; however, lower detection frequencies and more limited spatial distributions reduced the feasibility of robust species-specific analyses for those taxa (Fig 1).

Mallard detections were distributed across 305 locations, with high concentrations in the Pacific Northwest, Upper Midwest, Great Plains, and Northeast. Canada geese were detected across 500 locations, with high numbers in the Northeast, Upper Midwest, Pacific Coast, and Interior West. Green-winged teal detections were concentrated in the Southeast, with moderate detections across the Pacific Northwest, Pacific Coast, South, and Great Plains. Snow geese were found across 314 locations, with high detections in the South and moderate detections in the Midwest, Great Plains, and Northeast.

### Interpolated HPAI H5 detection intensity across waterfowl species

**Fig 2:**
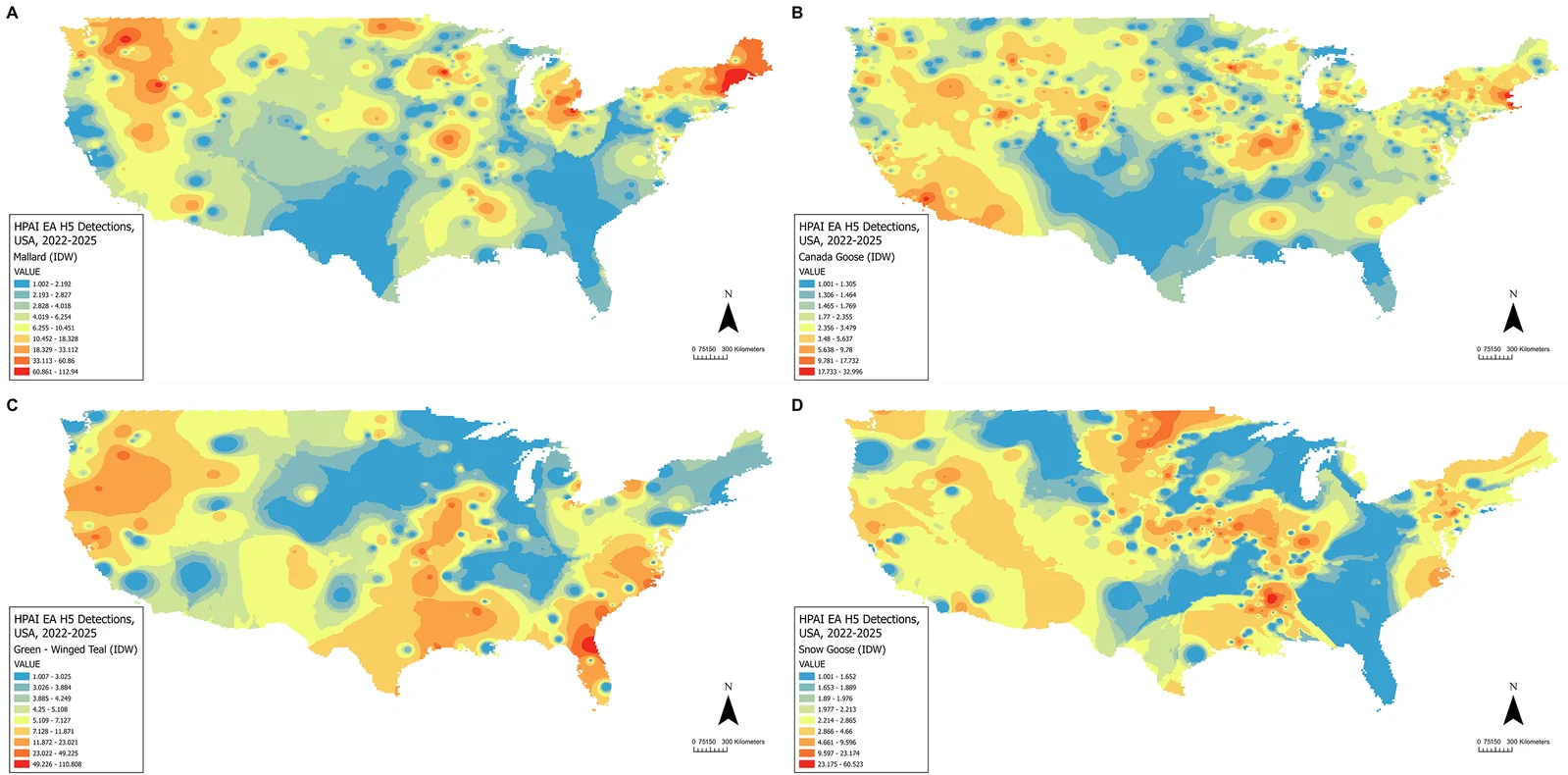
Spatial interpolation of highly pathogenic avian influenza (HPAI) H5 detections in wild waterfowl species using inverse distance weighting. (A) Mallard, (B) Canada goose, (C) Green-winged teal, and (D) Snow Goose. High detection areas are shown in red, medium detection areas in yellow tones, and low detection areas in blue.

Spatial interpolation identified distinct high-intensity HPAI detection zones (“hotspots”) that varied by vector species (Fig 2). Mallard detections were concentrated in the Northeast (New York, New England) and additional elevated areas in the Upper Midwest (Minnesota, Wisconsin, Michigan) and the Pacific Northwest (western Washington, Oregon). Low intensities were widespread across the Intermountain West, Southwest, and the central and southern Great Plains. High detection intensities for Canada goose were centered in the Upper Midwest (Minnesota, Wisconsin, Michigan) with additional elevated areas in the Northeast (New York, New England) and the Pacific Coast (California). Low intensities dominated the Intermountain West, Southwest, and much of the central and southern Great Plains. The highest intensities for Green-winged teal detections were concentrated in the Southeast, almost entirely in Florida. Moderate‑to‑elevated values extended across the South (Louisiana, Mississippi, Arkansas, Missouri) and the Pacific Northwest (Oregon, Washington). Low intensities were widespread across the northern and central United States, including the Upper Midwest, Northern Great Plains, and Intermountain West. High intensities for Snow goose were centered in the South, particularly Mississippi, with elevated zones extending through the Central Midwest and Northern Great Plains (Missouri, North Dakota, South Dakota) and into the Northeast (Pennsylvania). Low intensities were broadly distributed across the Intermountain West, Southwest, and large portions of the Great Plains.

### Space-time clustering of HPAI H5 among wild waterfowl

The space-time permutation scan statistic identified several significant space-time clusters for each wild bird species, where observed detections exceeded expected levels (O/E > 1) (Table 1).

**Table 1:** Space-time clusters of highly pathogenic avian influenza (HPAI) H5 detections in major wild bird species, United States, 2022–2025^a^.

Species	Cluster	Center coords (Lat, Long)	Radius (km)	Time frame	Cases	Expected cases	Observed / Expected	Test statistic	P value
**Mallard**	1	44.1658, −70.2065	150.38	2025−02–2025−03	222	48	4.63	171.51	1.00E-17
	2	44.0346, −94.0670	106.53	2025-04-01 to 2025-09-30	97	6.73	14.42	170.03	1.00E-17
	3	45.9373, −108.2744	769.72	2023−10–2023−11	186	41.24	4.51	139.22	1.00E-17
	4	43.8333, −83.0190	193.12	2025−10	103	11.79	8.73	133.48	1.00E-17
	5	35.5697, −93.4602	641.37	2024−12–2024−12	112	16.63	6.73	119.84	1.00E-17
	6	45.9950, −123.6557	44.19	2022−11	39	1.37	28.57	93.35	1.00E-17
	7	35.5178, −78.3657	830.49	2022−01–2022−03	72	15.54	4.63	54.51	1.00E-17
**Canada goose**	1	43.9150, −121.2281	802.89	2022−05–2022−09	117	31.06	3.77	71.81	1.00E-17
	2	42.6732, −70.9524	0	2024−03–2024−06	27	1.38	19.54	54.86	1.00E-17
	3	40.7326, −73.5862	299.32	2025−06–2025−08	118	40.59	2.91	50.61	1.00E-17
	4	39.8932, −94.4047	250.85	2025−12–2026−01	37	5.81	6.37	37.63	3.80E-15
	5	41.4041, −89.5286	162.35	2025−01	15	0.5	30.16	36.67	1.10E-14
	6	40.6664, −105.4611	448.39	2022−12–2023−02	56	14.69	3.81	34.20	1.90E-13
	7	39.6915, −83.8899	396.17	2025−09–2025−11	55	18.4	2.99	24.09	1.80E-08
**Green-winged teal**	1	30.1213, −93.8939	72.72	2022−11	49	6.19	7.92	59.25	1.00E-17
	2	34.6146, −78.5637	233.6	2022−01–2022−02	34	2.64	12.88	55.90	1.00E-17
	3	36.7152, −95.9044	170.36	2024−11	34	3.53	9.64	46.92	1.00E-17
	4	41.8506, −103.7080	683.02	2023−10	36	4.63	7.77	42.80	1.00E-17
	5	39.1693, −90.6676	75.91	2023−11	24	1.84	13.04	39.65	1.00E-17
	6	30.4580, −84.2779	311.48	2025−12	53	12.99	4.08	35.13	6.30E-15
	7	42.6864, −121.6501	0	2022−11	23	2.22	10.37	33.18	5.90E-14
	8	39.5981, −122.3919	109.77	2024−02	14	0.57	24.42	31.37	4.80E-13
	9	43.6251, −116.7093	0	2024−07	10	0.17	58.29	30.86	8.60E-13
	10	39.5151, −92.9626	0	2022−10–2022−11	20	3.06	6.53	20.69	1.10E-07
**Snow goose**	1	48.6855, −99.2457	401.73	2022−04–2022−05	89	13.49	6.60	95.12	1.00E-17
	2	38.8345, −81.6748	650.02	2025−01–2025−06	102	29.55	3.45	56.47	1.00E-17
	3	41.5779, −96.6540	388.03	2022−03	89	28.59	3.11	42.41	1.00E-17
	4	33.7971, −90.8806	284.26	2022−12–2024−12	182	88.39	2.06	42.38	1.00E-17
	5	38.0735, −122.7236	129.75	2024−02–2024−03	17	0.65	26.21	39.30	1.00E-17

^a^For each wild waterfowl species type, Cluster 1 represents the primary cluster (i.e., the most likely cluster with the highest test statistic), followed by secondary clusters in decreasing order of importance. Clusters were identified using the retrospective space-time permutation scan statistic with a scanning window maximum of up to 50% of the background population at risk and 50% of the study period. Statistical significance was evaluated using 999 Monte Carlo replications, with clusters considered significant at p ≤ 0.05. Reported radii represent the spatial extent of the detected clusters, and coordinates indicate the centroid of each identified cluster.

**Fig 3:**
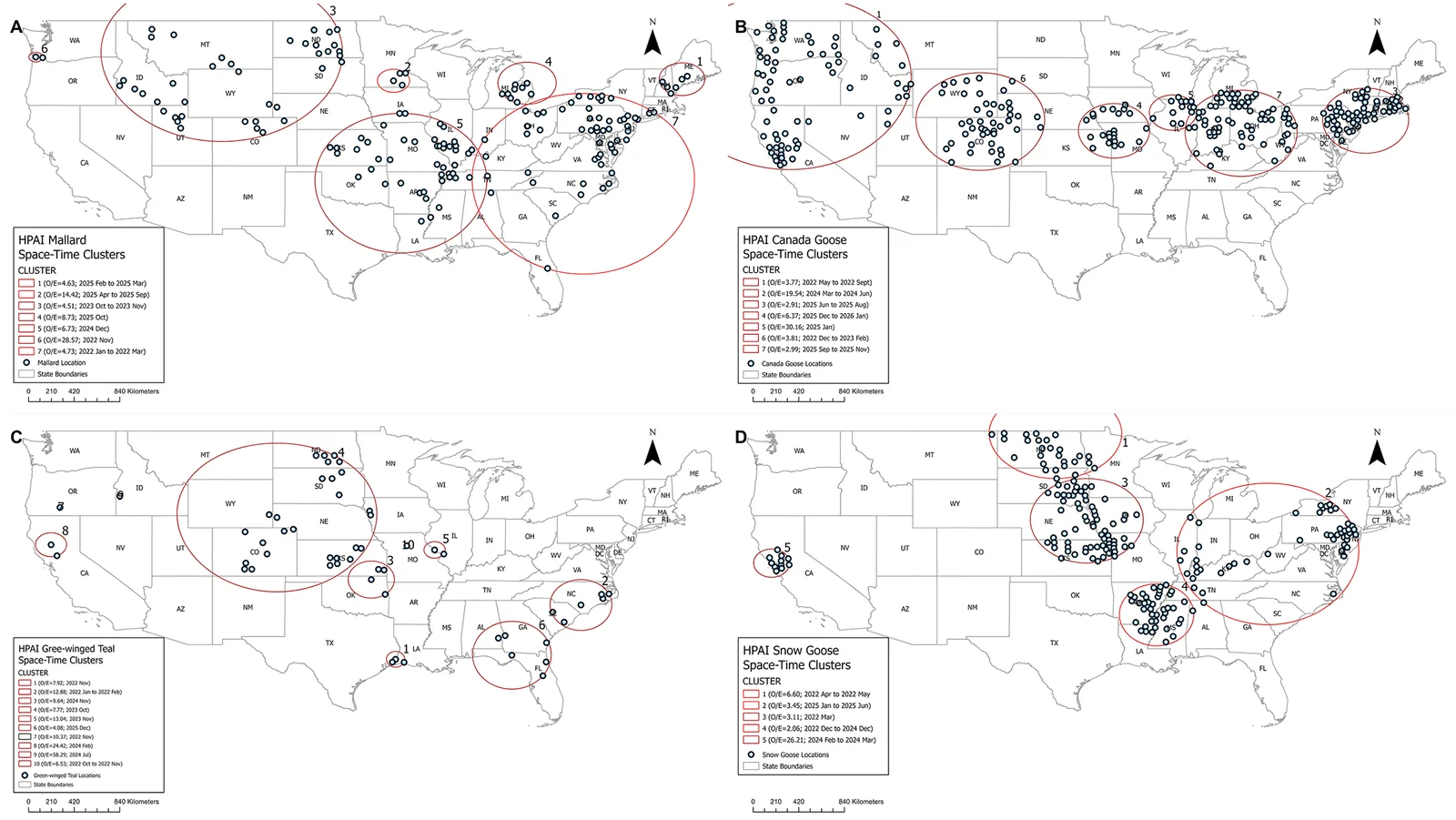
Results of space-time permutation models for highly pathogenic avian influenza (HPAI) H5 detections in wild waterfowl species across the U.S., 2022-2025. (A) Mallard, (B) Canada goose, (C) Green-winged teal, and (D) Snow Goose. The analysis used a cylindrical scanning window with 50% of the population and 50% of the study period, highlighting significant space-time clusters of higher-than-expected detections. Significant at p ≤ 0.05, using 999 Monte Carlo simulations.

These clusters varied in magnitude, duration, and spatial extent, reflecting periods of elevated detection intensity (Fig 3, Table 1). Seven clusters were identified for Mallard. The primary cluster (O/E = 4.63, Feb-Mar 2025) occurred in the Northeast, while a high-intensity cluster (O/E = 14.42) centered on Minnesota during spring to early fall 2025. Other clusters were in Michigan (O/E = 8.73, Oct 2025) and the Pacific Northwest (O/E = 8.57, Nov 2022). Canada goose had seven clusters. The primary cluster (O/E = 3.77, May-Sept 2022) spanned the West Coast and Northwest. A high-intensity cluster (O/E = 30.16) occurred in the Midwest (Jan 2025). Additional clusters were in the Northeast and Pacific Northwest. Ten clusters were identified for Green-winged teal, with the primary cluster (O/E = 7.92) in Louisiana (Nov 2022). A high-intensity spike (O/E = 58.29, Jul 2024) was observed in Oregon. Other clusters spanned the Southeast, Midwest, and West Coast. Five Snow goose clusters were found, including a primary cluster (O/E = 6.60, Apr–May 2022) in the Northern Plains. A long-duration cluster (O/E = 2.06) spanned two years in the South, while the strongest cluster (O/E = 26.21) was centered in California during late winter 2024.

When comparing wild waterfowl species, Mallard and Canada goose demonstrated broad, recurrent clusters spanning multiple flyways, whereas green-winged teal exhibited short-duration, localized, high-intensity bursts. Snow geese displayed fewer clusters but sustained low-to-moderate elevation across extended periods.

### Distribution of H5 HPAI H5 outbreaks in commercial poultry operations

**Fig 4:**
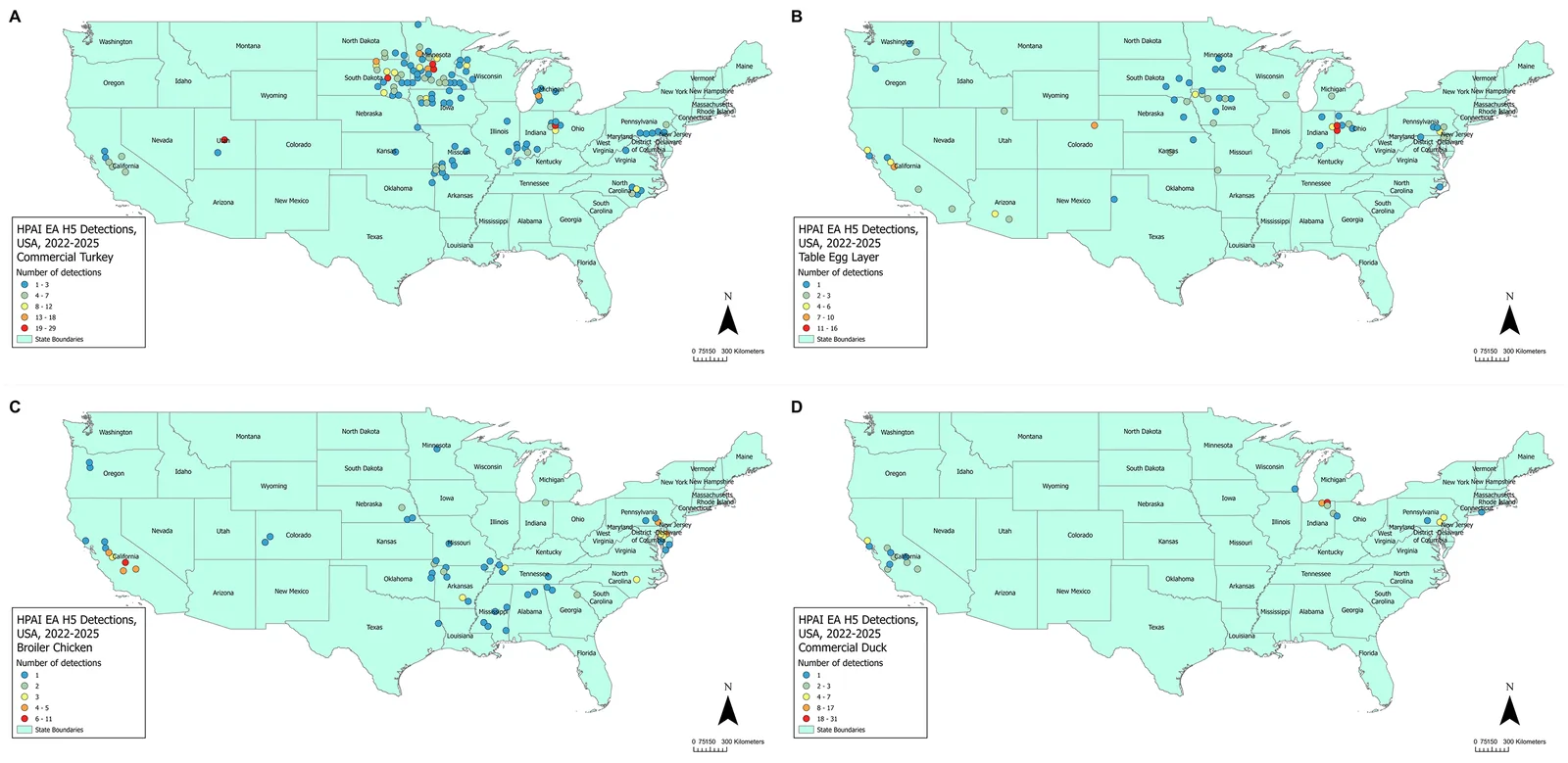
Distribution of highly pathogenic avian influenza (HPAI) H5 outbreaks in commercial poultry farms across the U.S., 2022–2025. (A)Turkey, (B)Table egg layer, (C) Broiler Chicken, and (D) Duck. Divergent colors are used, with red indicating high and blue indicating low outbreak number areas.

Turkey operations had 532 outbreaks across 121 locations between February 8^th,^ 2022, and January 16^th,^ 2026 (Fig 4). A high number of detections were in the Upper Midwest (Minnesota and South Dakota), the Great Lakes (Michigan, Ohio), and the Intermountain West (Utah). Table‑egg layer operations had 148 outbreaks across 55 locations between February 22^nd,^ 2022, and January 6^th^, 2026. A high number of detections were in the Midwest (Ohio, Indiana, Iowa), the Mountain West (Colorado, Arizona), the Mid‑Atlantic (Pennsylvania, Delaware), and the West Coast (California). Broiler chicken operations exhibited 99 outbreaks across 54 locations between February 12^th,^ 2022, and January 13^th^, 2026. A high number of detections were in the West Coast (California), the Mid‑Atlantic (Pennsylvania, Delaware), and the South (Arkansas, Tennessee). Commercial duck operations had 89 outbreaks across 20 locations between April 8^th^, 2022, and December 29^th^, 2025. A high number of detections were in the Midwest (Indiana, Ohio), the Mid‑Atlantic (Pennsylvania), and the West Coast (California).

### Space-time clustering of HPAI H5 among commercial poultry operations

**Fig 5:**
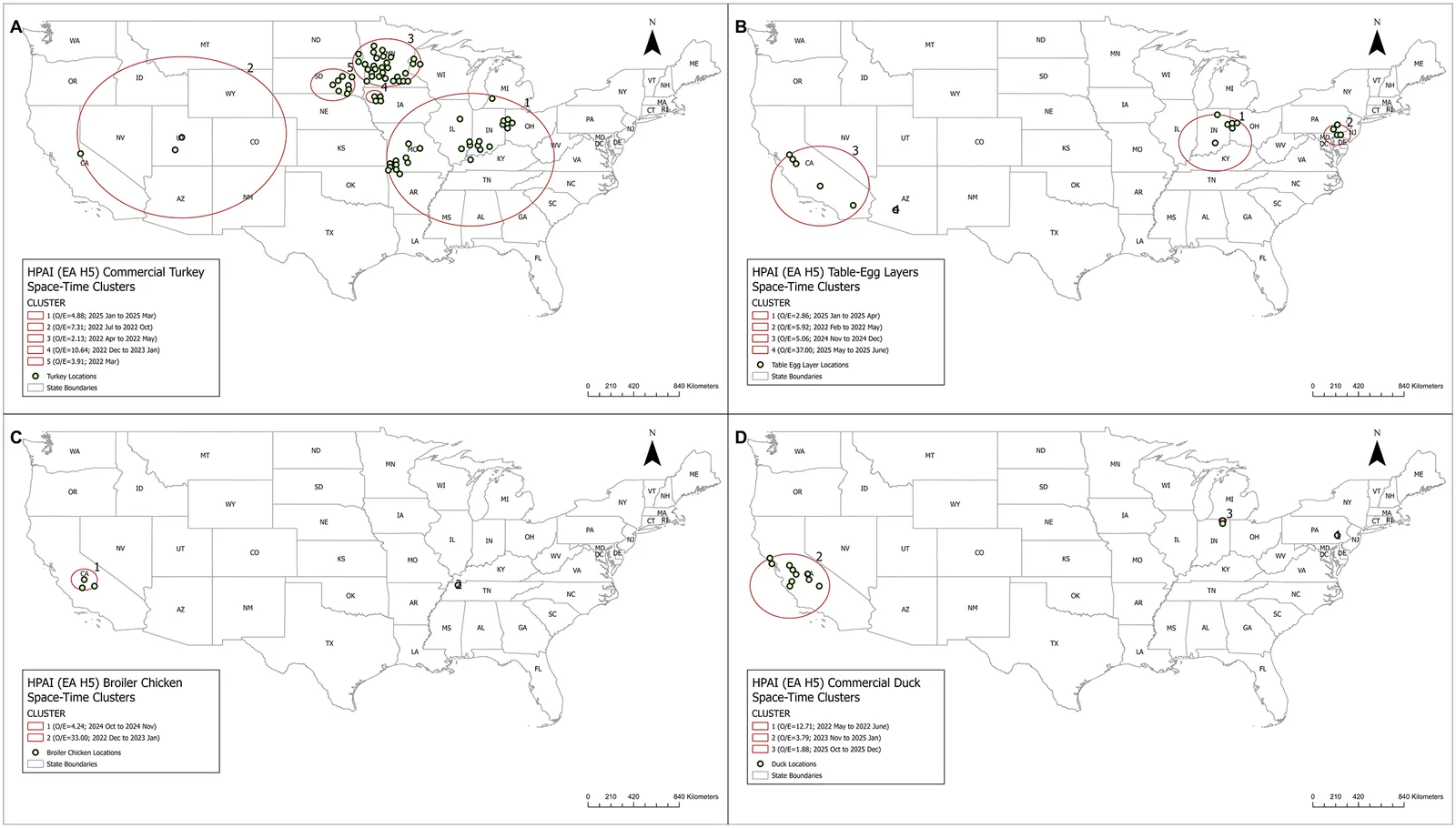
Space-time permutation model results for highly pathogenic avian influenza (HPAI) H5 outbreaks in poultry species across the U.S., 2022-2025. (A)Turkey, (B)Table egg layer, (C) Broiler Chicken, and (D) Duck. The analysis used a cylindrical scanning window with 50% of the population and 50% of the study period, highlighting significant space-time clusters of higher-than-expected outbreaks. Significant at p ≤ 0.05, using 999 Monte Carlo simulations.

Across commercial poultry operations, multiple significant space-time clusters of HPAI H5 were identified, varying by production type (Fig 5, Table 2).

**Table 2:** Space-time clusters of highly pathogenic avian influenza (HPAI) H5 outbreaks across major U.S. poultry sectors, 2022–2025^a^.

Poultry type	Cluster	Center coords (Lat, Long)	Radius (km)	Time frame	Cases	Expected cases	Observed/ expected	Test statistic	P value
**Turkey**	1	37.5184, −87.6832	610.45	2025−01–2025−03	64	13.11	4.88	53.16	1.00E-17
	2	39.3739, −111.5763	741.57	2022−07–2022−10	22	3.01	7.31	25.13	2.70E-11
	3	45.5521, −94.6131	218.98	2022−04–2022−05	60	28.22	2.13	14.50	2.20E-05
	4	42.7356, −95.6238	57.51	2022−12–2023−01	8	0.75	10.64	11.72	7.70E-04
	5	43.7181, −99.0809	146.15	2022−03	14	3.58	3.91	8.78	0.029
**Table egg layer**	1	38.9064, −86.0375	260.21	2025−01–2025−04	36	12.58	2.86	16.58	1.20E-08
	2	39.5712, −75.9407	93.92	2022−02–2022−05	12	2.03	5.92	11.72	2.10E-05
	3	35.3426, −118.7301	366.47	2024−11–2024−12	13	2.57	5.06	11.04	5.90E-05
	4	33.3490, −112.4915	0	2025−05–2025−06	4	0.11	37.00	10.60	1.10E-04
**Broiler chicken**	1	36.7582, −119.6493	96.56	2024−10–2024−11	12	2.83	4.24	8.62	8.90E-04
	2	36.2983, −88.7178	0	2022−12–2023−01	3	0.091	33.00	7.62	0.0047
**Commercial duck**	1	40.4163, −75.9260	0	2022−05–2022−06	7	0.55	12.71	11.59	2.00E-06
	2	36.2171, −121.2388	295.32	2023−11–2025−01	17	4.48	3.79	11.12	4.20E-06
	3	41.6417, −85.4261	27.02	2025−10–2025−12	32	17.06	1.88	6.86	0.0032

^a^For each poultry type, Cluster 1 represents the primary cluster (i.e., the most likely cluster with the highest test statistic), followed by secondary clusters in decreasing order of importance. Clusters were identified using the retrospective space-time permutation scan statistic with a scanning window maximum of up to 50% of the background population at risk and 50% of the study period. Statistical significance was evaluated using 999 Monte Carlo replications, with clusters considered significant at p ≤ 0.05. Reported radii represent the spatial extent of the detected clusters, and coordinates indicate the centroid of each identified cluster.

Commercial turkeys exhibited five clusters. Cluster 1 (O/E = 4.88; Jan–Mar 2025) occurred in the Upper Midwest. Cluster 2 (O/E = 7.31; Jul-Oct 2022) spanned the Upper Midwest and Northern Plains. Cluster 3 (O/E = 2.13; Apr-May 2022) reflected early spring activity in the central United States. Clusters 4 (O/E = 10.64; Dec 2022-Jan 2023) and 5 (O/E = 3.91; Mar 2022) represented overlapping winter hotspots in the Upper Midwest. Table‑egg layers showed four clusters. Cluster 1 (O/E = 2.86; Jan-Apr 2025) occurred in Indiana and Ohio. Cluster 2 (O/E = 5.92; Feb-May 2022) was centered in Pennsylvania and Delaware. Cluster 3 (O/E = 5.06; Nov-Dec 2024) occurred in California, and Cluster 4 (O/E = 37.00; May-Jun 2025) represented an intense, short‑duration cluster in Arizona. Commercial broilers had two clusters. Cluster 1 (O/E = 4.42; Feb-Apr 2024) occurred in California, while Cluster 2 (O/E = 6.89; Nov-Dec 2023) was centered in Tennessee, each reflecting localized periods of elevated activity. Commercial ducks exhibited three clusters. Cluster 1 (O/E = 12.71; May–Jun 2022) occurred in Pennsylvania. Cluster 2 (O/E = 3.79; Nov 2023–Jan 2025) was centered in California, and Cluster 3 (O/E = 1.88; Oct–Dec 2025) occurred in Indiana, marking a late‑year cluster of lower relative intensity.

Spatiotemporal overlap between the primary turkey and layer outbreak clusters in the Midwest (Ohio and Indiana) was observed in early 2025. In addition, spatiotemporal overlap between outbreaks in table egg layers (Cluster 3), broiler chickens (Cluster 1), and commercial ducks (Cluster 2) in the Pacific Coast region (California) during the fall of 2024 was identified. These overlaps occurred in high-poultry-production-density regions near major migratory flyways.

### Spatiotemporal overlap of HPAI H5 detections in wild waterfowl and commercial poultry farm outbreaks

Several spatiotemporal associations between wild waterfowl detections and poultry outbreaks were identified (Table 3).

**Table 3:** Putative HPAI H5 spillover events inferred from overlapping significant wild waterfowl and commercial poultry space-time clusters, United States, 2022-2025.

Event	Wild waterfowl cluster(s)	Wild waterfowl period	Poultry cluster(s)	Poultry period	Temporal relationship	Geographic region/Flyway	Supporting statistical evidence
**1**	Mallard C7	Jan-Mar 2022	Table-egg layer C2; Commercial duck C1	Feb-May 2022; May-Jun 2022	Wild-bird detections preceded poultry outbreaks by 1–4 months and overlapped outbreak onset	Northeast U.S. (Atlantic Flyway)	Mallard: TS = 54.51, p < 0.001; Egg layer: TS = 11.72, p < 0.001; Commercial Duck: TS = 11.59, p < 0.001
**2**	Snow goose C3; Snow goose C1	Mar 2022; Apr-May 2022	Turkey C5; Turkey C3	Mar 2022; Apr-May 2022	Concurrent space-time clusters during spring migration	Upper Midwest (Mississippi Flyway)	Snow goose C3: TS = 42.41, p < 0.001; Snow goose C1: TS = 95.12, p < 0.001; Turkey C5: TS = 8.78, p = 0.029; Turkey C3: TS = 14.50, p < 0.001
**3**	Canada goose C1	May-Sep 2022	Turkey C2	Jul-Oct 2022	Poultry outbreaks occurred during latter portion of wild-bird cluster period	Pacific and Mountain West (Pacific Flyway)	Canada goose: TS = 71.81, p < 0.001; Turkey: TS = 25.13, p < 0.001
**4**	Canada goose C6	Dec 2022-Feb 2023	Turkey C4	Dec 2022-Jan 2023	Complete temporal overlap during winter outbreak period	Upper Midwest and Mountain West	Canada goose: TS = 34.20, p < 0.001; Turkey: TS = 11.72, p < 0.001
**5**	Snow goose C5; Green-winged teal C8	Feb-Mar 2024; Feb 2024	Commercial duck C2; Broiler C1; Table-egg layer C3	Nov 2023-Jan 2025; Oct-Nov 2024; Nov-Dec 2024	Wild-bird detections occurred within the broader period of sustained poultry activity in the Pacific region	Pacific Coast (Pacific Flyway)	Snow goose: TS = 39.30, p < 0.001; Teal: TS = 31.37, p < 0.001; Duck: TS = 11.12, p < 0.001; Broiler: TS = 8.62, p < 0.001; Layer: TS = 11.04, p < 0.001
**6**	Snow goose C2; Canada goose C5	Jan-Jun 2025; Jan 2025	Turkey C1; Table-egg layer C1	Jan-Mar 2025; Jan-Apr 2025	Direct temporal overlap, with poultry clusters occurring entirely within wild-bird cluster period	Midwest/Ohio Valley (Mississippi Flyway)	Snow goose: TS = 56.47, p < 0.001; Canada goose: TS = 36.67, p < 0.001; Turkey: TS = 53.16, p < 0.001; Layer: TS = 16.58, p < 0.001
**7**	Canada goose C7; Mallard C4	Sep-Nov 2025; Oct 2025	Commercial duck C3	Oct-Dec 2025	Wild-bird detections preceded and overlapped poultry outbreaks	Upper Midwest (Mississippi Flyway)	Canada goose: TS = 24.09, p < 0.001; Mallard: TS = 133.48, p < 0.001; Duck: TS = 6.86, p = 0.003

^a^Putative spillover events were defined as significant wild waterfowl and commercial poultry space-time clusters exhibiting spatial proximity and temporal overlap or succession. Events are presented as epidemiologic evidence consistent with potential spillover; direct transmission cannot be confirmed without viral genomic sequencing and phylogenetic analyses.

The first potential spillover event occurred during winter-spring 2022 in the Northeast U.S. (Atlantic Flyway), where detections in Mallards (Cluster 7; January-March 2022) preceded outbreaks in table-egg layer (Cluster 2; February-May 2022) and commercial duck farms (Cluster 1; May-June 2022). The second event occurred during spring 2022 in the Upper Midwest (Mississippi Flyway), where snow goose detections (Cluster 3; March 2022; Cluster 1; April-May 2022) coincided with outbreaks in commercial turkey farms (Cluster 5; March 2022 and Cluster 3; April-May 2022). The third event occurred in the Pacific and Mountain West regions (Pacific Flyway), where detections in Canada goose (Cluster 1; May-September 2022) coincided with turkey outbreaks (Cluster 2; July-October 2022). The fourth event occurred during winter 2022–2023 in the Upper Midwest, where Canada goose detections (Cluster 6; December 2022-February 2023) in the Midwest and Mountain West overlapped with commercial turkey outbreaks (Cluster 4; December 2022-January 2023). The fifth event occurred during the fall-winter of 2025 in the Pacific region (Pacific Flyway), where snow goose (Cluster 5; February-March 2024) and green-winged teal (Cluster 8; February 2024) detections coincided with duck outbreaks (Cluster 2; November 2023–January 2025). Additionally, outbreaks in broiler chickens (Cluster 1; October-November 2024) and table-egg layers (Cluster 3; November-December 2024) were seen in this region. The sixth event occurred during the winter-spring of 2025 in the Midwest (Mississippi Flyway), where snow goose (Cluster 2; January-June 2025) and Canada goose (Cluster 5; January 2025) detections coincided with outbreaks in turkey (Cluster 1; January-March 2025) and table-egg layer (Cluster 1; January-April 2025) farms. The last spillover event occurred during the fall-winter of 2025 in the Upper Midwest, where Canada goose (Cluster 7; September-November 2025) and mallard (Cluster 4; October 2025) detections overlapped with outbreaks in commercial duck farms (Cluster 3; October-December 2025).

## Discussion

This study analyzed surveillance data on wild waterfowl HPAI H5 detections and commercial poultry outbreaks from February 2022 until early January 2026. Disease mapping and space-time permutation models analyzed wild waterfowl detections and commercial poultry outbreaks and identified distinct regional and temporal patterns. The spatiotemporal analysis identified seven cluster overlaps between wild waterfowl HPAI H5 detections and commercial poultry HPAI H5 outbreaks, suggesting potential spillover events at the wild waterfowl-commercial poultry farm interface. These events can be categorized into four phases. The first phase covered winter-spring 2022, corresponding to the initial incursion of the virus via the Atlantic Flyway [17], and the amplification of the virus in Mallards before the potential spillover to commercial ducks and table-egg layers (Event 1). The second phase included the spring-fall 2022 period, when the virus expanded westward (Events 2 & 3) via the Mississippi Flyway, driven by the high viral environmental load from migration of Snow goose and Canada goose, where commercial turkey farms were affected. Phase 3 included the period between winter 2022 and 2024, where persistence of the virus in Canada goose and outbreaks in turkey farms occurred in the Midwest (Event 4). This phase also included the fourth event in the Pacific region, with a prolonged outbreak in commercial ducks showing a persistence of the virus (fall 2023 to winter 2025) and short localized outbreaks in broiler chickens and table egg layers in fall 2024. In this region, space-time outbreak clusters were identified in Snow goose and Green-winged teal during winter 2024, suggesting an expansion of the host range. Finally, Phase 4 included the winter of 2025, when the virus resurged in the Midwest (Events 6 & 7), suggesting a cyclical reinfection of the Mississippi Flyway, and high viral load and detections in Canada goose, Snow goose, and Mallard, and large outbreaks in turkey, table egg, and commercial duck farms, mirroring Phase 1 and 2 in its extent and severity.

This study identified species-specific spatiotemporal patterns of HPAI H5 detections. Canada goose HPAI H5 detection clusters repeatedly coincided with commercial turkey outbreak clusters across the Midwest and Great Plains (Events 3, 4, 6, and 7), suggesting a potential epidemiologic linkage that warrants further investigation using viral genomic and phylogenetic data. Canada goose populations are abundant in agricultural fields and in proximity to poultry farms [24], graze on farm peripheries and overwinter near human habitat, and might act as a local environmental reservoir and a bridge species, linking the gap between seasonal migration waves, allowing the virus to overwinter. In addition, in late 2025 (Event 7), the overlap of Canada goose detections (Cluster 7) with outbreaks in commercial duck farms suggests a transmission shift.

Our analysis of Event 2 (Spring 2022) and Event 6 (Winter-Spring 2025) identified a link between detections in Snow geese and outbreaks in commercial turkey operations. Snow geese are an effective HPAI H5 transmitter [25], migrating in large flocks, which facilitates rapid viral amplification. The detection of HPAI H5 in Snow geese (Clusters 1 and 3) immediately preceding outbreaks in Midwestern turkey farms suggests that high environmental viral loads might have facilitated viral transmission to turkey farms. The recurrence of this specific Goose-to-Turkey potential spillover pathway in 2025 in the Midwest underlines the vulnerability of turkey operations, especially during the winter and fall migration seasons, and calls for enhanced biosecurity programs [26].

Mallard detections were associated with outbreaks in commercial duck and layer farms (Events 1 and 7), suggesting that they might act as maintenance hosts. Previous studies described that Mallards can be infected and shed high viral loads, but they remain asymptomatic or exhibit only mild clinical signs [27]. This phenomenon poses a high risk for the commercial duck sector, where proximity to wild reservoirs can facilitate viral amplification and transmission if farm biosecurity breaches occur. The recurrence of this pattern in the Upper Midwest in late 2025 (Event 7) underscores the long-term risk posed by resident or semi-migratory Mallard populations overwintering near poultry production sites.

Within commercial poultry, turkeys and table-egg layers exhibited the most consistent and overlapping outbreak clusters, particularly in the Midwest. The recurrent co-localization of turkey and layer outbreaks in this region implies that these regions within major migratory flyways are at heightened risk and could function as amplification zones following wild-bird introductions. The Mississippi Flyway, home to high densities of commercial poultry in the Midwest, saw significant spillover events during the spring and fall migrations of 2022 and 2025. On the other hand, broiler and commercial duck clustering appeared more episodic or geographically focal.

Our analysis suggests that the current HPAI H5 strain, especially the 2.3.4.4b clade, can persist in the environment and can be sustained by the local wild waterfowl populations, posing a continuous risk to poultry operations [11]. The current outbreak has evolved from a seasonal, migratory introduction (2022) into a persistent, multi-vector endemic system (2025), with Snow Geese and Canada Geese emerging as the primary bridge vectors for commercial turkey flocks. Rather than a single introduction wave, the findings of our study support a pattern of repeated seasonal spillover events [28], followed by regional amplification within high-density poultry production systems, especially turkey and table egg layer farms, suggesting a possible spread via fomites.

The observation that HPAI H5 detections in wild waterfowl preceded poultry outbreaks suggests that HPAI surveillance in wild waterfowl could provide an early warning period to implement enhanced biosecurity measures on poultry farms. During periods of increased HPAI activity in wild birds, poultry producers should reinforce biosecurity measures [29] such as excluding wild birds from poultry facilities, protecting feed and water sources, restricting farm access, using dedicated clothing and footwear, and ensuring effective cleaning and disinfection of vehicles and equipment. Such targeted interventions may reduce the risk of virus introduction at the wild waterfowl-poultry interface [15].

Risk-based resource allocation, considering the location, poultry production types, and wild bird types, may improve outbreak detection and response efficiency compared with uniform national approaches. The recurrent overlap between turkey and layer HPAI H5 outbreak clusters indicates that multi-commodity coordination in regions with active wild-bird pressure could improve outbreak control. Additionally, farm biosecurity programs should be updated and synchronized across poultry sectors, considering local disease pressures, and they are cost-effective in preventing outbreaks [30]. Furthermore, implementing spatial-temporal cluster detection methods into surveillance systems [31] and integrating wild bird and poultry HPAI H5 surveillance could improve outbreak preparedness and control. Finally, linking migratory waterfowl detection intensity with poultry density maps could inform animal health authorities to anticipate risk and proactively implement prevention and control programs [14].

Before implementing the study results, a few limitations should be noted. First, the analyses were restricted to the four most frequently detected waterfowl species, which represented nearly 40% of all wild waterfowl HPAI H5 detections and provided sufficient observations for space-time cluster analysis; however, additional waterfowl species may also contribute to HPAI H5 transmission dynamics, and future studies should assess them. In addition, HPAI surveillance in the U.S. is conducted through a comprehensive national monitoring system targeting multiple wild bird species and migratory flyways; however, variation in surveillance intensity between regions, species, and seasons may have influenced detection probabilities and consequently the identification of some space-time clusters. Also, because outbreak counts rather than incidence rates adjusted for the underlying poultry population at risk were analyzed, some detected poultry clusters may partially reflect the geographic distribution of poultry operations and production density. Finally, the absence of viral genomic and phylogenetic data precluded direct confirmation of wild bird-to-poultry transmission pathways.

## Conclusion

This study identified distinct spatiotemporal patterns of HPAI H5 detections in major wild waterfowl species and HPAI H5 outbreaks in commercial poultry sectors across the United States between 2022 and 2025. The observed clustering patterns supported our hypothesis that wild waterfowl detections and poultry outbreaks would exhibit significant space-time clustering and spatial-temporal correspondence within major migratory flyways. Moreover, these associations varied among waterfowl species and poultry production sectors. Several putative spillover events were inferred from significant spatiotemporal associations between wild waterfowl and poultry clusters. The recurring observation that wild waterfowl clusters preceded or overlapped poultry outbreak clusters highlights the potential value of integrated wild bird surveillance as an early warning component of poultry health management. Collectively, these findings support risk-based surveillance and enhanced biosecurity measures tailored to local wild bird populations, migratory flyways, and poultry production systems.
