# Supplementary material for: Spatiotemporal clustering of highly pathogenic avian influenza (HPAI) H5N1 at the wild waterfowl-poultry interface: Vector-specific spillover risks in the U.S., 2022–2025
Source: PLoS One. 2026 Sep 23;21(9):e0345354. doi: 10.1371/journal.pone.0345354 (PMC13600479; doi:10.1371/journal.pone.0345354)
Supplement: Supplementary file 1 [file pone.0345354.s002.docx]

Dear Dr. Varga,

Thank you for submitting your manuscript to PLOS ONE. After careful consideration, we feel that it has merit but does not fully meet PLOS ONE’s publication criteria as it currently stands. Therefore, we invite you to submit a revised version of the manuscript that addresses the points raised during the review process.

The reviewers cited several shortcomings that make the article unsuitable for acceptance to publish in the present form. These short are widespread across the various sections of the paper but mostly in the methodological protocol, and as a result of which the results presented and discussed, and from which conclusions are drawn are largely in discordance.

In particular:

- Temporal and spatial variations on bird/poultry observations is interpreted to imply infection risk spillover, without demonstrated epidemiological evidence through analyses of viral sequencing

**Response:** We addressed these concerns. Please see my response to the reviewers below.

- Potential biases in regional (state to state) levels of variation in poultry production intensity or surveillance in relation to risk infection, is not accounted for in the analyses

**Response:** Limitations of the analyses were included.

- Need for more clearer and transparent presentation of results in tabular form an in a more complete/comprehensive way

**Response:** A new table was generated to streamline the comparison between wild waterfowl and poultry outbreaks.

- The choice of the range of study species of wild birds is not justified in the Methods (i.e. why these specie in particular yet they are not necessarily the most vulnerable, or the most significant risk carriers?)

**Response:** The rationale for choosing the 4 wild waterfowl species was included.

Many more specific issues are highlighted by the reviewers and need to be addressed in point by point form in a separate document to accompany the major revision of the paper

**Response:** I addressed all the suggestions and comments point by point below.

Two other major outstanding drawbacks of the paper are:

1. The very small number of literature cited literature (24). For a subject with such a large proliferation of research in the last 5 years, this small number of citations clearly indicates serious in adequacy in literature review and subliminal justification f the gap that the study serves to fill. It might actually turn out that this topic has already been researched and published in recent times, and so it is necessary to conduct a thorough literature search (at least 60 % no older than 10 years) in the Introduction section to more clearly demonstrate novelty for your particular topic. The introduction needs a lot more expansion to provide a broader background information to the study. AS one reviewer has suggested, elements such as genetic drifts and the transmission mode for the virus, must be mentioned as early as possible

**Response:** Thank you for this comment. I agree that the Introduction would benefit from additional background and a more comprehensive review of the rapidly expanding HPAI literature. In response, we expanded the Introduction and incorporated additional recent references addressing the emergence and spread of HPAI viruses, avian influenza transmission pathways, public health risk, and on-farm biosecurity practices.

We also clarified the novelty of the present study. Although numerous studies have examined HPAI occurrence in wild birds, poultry outbreaks, viral evolution, surveillance programs, and transmission pathways, my literature review did not identify previous studies that conducted species-specific national-scale space-time cluster analyses of major wild waterfowl species and commercial poultry sectors and subsequently evaluated their spatiotemporal associations across migratory flyways in the United States. The Introduction was revised to more to articulate this knowledge gap and the rationale for the study.

2. At the end of the Introduction section, there is no clear indication of what hypotheses were tested to justify the modeling, or at least a statement of what the study expected to find. This is necessary and should be clearly to bot the knowledge gap addressed and the specific objectives pursued, as well as reflected in the Conclusion, to show whether those hypotheses were ultimately proved or not.

**Response:** Thank you for your comment. We included the following in the Introduction: “We hypothesized that both wild waterfowl detections and poultry outbreaks would exhibit significant space-time clustering, and that clusters occurring within the same migratory flyways would demonstrate spatial and temporal associations indicative of potential epidemiological connectivity. Furthermore, we expected these associations to differ among waterfowl species and poultry production sectors.

We included the following in the Conclusion section: “The observed clustering patterns supported our hypothesis that wild waterfowl detections and poultry outbreaks would exhibit significant space-time clustering and spatial-temporal correspondence within major migratory flyways. “

For the revised paper, the authors must also adhere more closely to the journal’s forming rules according the Submission Guidelines, including font type and sizes, grammar and figure quality as well as sequential arrangement of the various  sections and numbering of in-text literature citations.

**Response:** Manuscript updated to follow the submission guidelines.

Journal Requirements:

When submitting your revision, we need you to address these additional requirements.

1. Please ensure that your manuscript meets PLOS ONE's style requirements, including those for file naming. The PLOS ONE style templates can be found at

https://journals.plos.org/plosone/s/file?id=wjVg/PLOSOne_formatting_sample_main_body.pdf and

<https://journals.plos.org/plosone/s/file?id=ba62/PLOSOne_formatting_sample_title_authors_affiliations.pdf>

**Response:** Manuscript updated to meet the style requirements.

2. We note that All Figures in your submission contain map/satellite images which may be copyrighted. All PLOS content is published under the Creative Commons Attribution License (CC BY 4.0), which means that the manuscript, images, and Supporting Information files will be freely available online, and any third party is permitted to access, download, copy, distribute, and use these materials in any way, even commercially, with proper attribution. For these reasons, we cannot publish previously copyrighted maps or satellite images created using proprietary data, such as Google software (Google Maps, Street View, and Earth). For more information, see our copyright guidelines: http://journals.plos.org/plosone/s/licenses-and-copyright.

We require you to either (1) present written permission from the copyright holder to publish these figures specifically under the CC BY 4.0 license, or (2) remove the figures from your submission:

**Response:** The maps were created by me, using GIS software, which does not require copyright. We included the following: “Disease maps and spatial statistical analyses were conducted in ArcGIS Pro 10.7.1 (Environmental Systems Research Institute, Inc., Redlands, CA, USA). All maps were generated in ArcGIS Pro 10.7.1 using county-level HPAI surveillance data and U.S. Census Bureau state boundary shapefiles.”

1. You may seek permission from the original copyright holder of All Figures to publish the content specifically under the CC BY 4.0 license.

We recommend that you contact the original copyright holder with the Content Permission Form (http://journals.plos.org/plosone/s/file?id=7c09/content-permission-form.pdf) and the following text:

“I request permission for the open-access journal PLOS ONE to publish XXX under the Creative Commons Attribution License (CCAL) CC BY 4.0 (http://creativecommons.org/licenses/by/4.0/). Please be aware that this license allows unrestricted use and distribution, even commercially, by third parties. Please reply and provide explicit written permission to publish XXX under a CC BY license and complete the attached form.”

Please upload the completed Content Permission Form or other proof of granted permissions as an ""Other"" file with your submission.

In the figure caption of the copyrighted figure, please include the following text: “Reprinted from [ref] under a CC BY license, with permission from [name of publisher], original copyright [original copyright year].”

**Response:** The figures do not require copyright. We included the following in the Methods section: “Disease maps and spatial statistical analyses were conducted in ArcGIS Pro 10.7.1 (Environmental Systems Research Institute, Inc., Redlands, CA, USA). All maps were generated in ArcGIS Pro 10.7.1 using county-level HPAI surveillance data and U.S. Census Bureau state boundary shapefiles.”

2. If you are unable to obtain permission from the original copyright holder to publish these figures under the CC BY 4.0 license or if the copyright holder’s requirements are incompatible with the CC BY 4.0 license, please either i) remove the figure or ii) supply a replacement figure that complies with the CC BY 4.0 license. Please check copyright information on all replacement figures and update the figure caption with source information. If applicable, please specify in the figure caption text when a figure is similar but not identical to the original image and is therefore for illustrative purposes only.

The following resources for replacing copyrighted map figures may be helpful:

USGS National Map Viewer (public domain): http://viewer.nationalmap.gov/viewer/

The Gateway to Astronaut Photography of Earth (public domain): http://eol.jsc.nasa.gov/sseop/clickmap/

Maps at the CIA (public domain): https://www.cia.gov/library/publications/the-world-factbook/index.html and https://www.cia.gov/library/publications/cia-maps-publications/index.html

NASA Earth Observatory (public domain): http://earthobservatory.nasa.gov/

Landsat: http://landsat.visibleearth.nasa.gov/

USGS EROS (Earth Resources Observatory and Science (EROS) Center) (public domain): http://eros.usgs.gov/#

Natural Earth (public domain): <http://www.naturalearthdata.com/>

**Response:** All these are not applicable to my manuscript as I made the maps myself in ArcGIS Pro.

3. If the reviewer comments include a recommendation to cite specific previously published works, please review and evaluate these publications to determine whether they are relevant and should be cited. There is no requirement to cite these works unless the editor has indicated otherwise.

**Response:** Not applicable.

**Reviewer #1**:

Varga C. investigated spatiotemporal clustering of HPAI (H5N1) at the wild waterfowl-poultry interface in the USA and considered farm biosafety strategies to contain these recurrent, vector-specific risks. The study offers a thorough analysis, making this article a good read for understanding these spillovers.
Below are the comments-

• Overall, this is a well-written research article on a worthy topic supported by good methodologies and results. Further improvements and revisions will undoubtedly enhance its overall quality.
• The title reflects the study well.
• The abstract aligns well with the study and conveys it effectively. Further updates in the abstract will definitely enhance its overall quality.

Comments:

• Line 34: In the conclusion section of the abstract, the author highlighted the critical importance of farm biosecurity strategies, which must be given more emphasis in the manuscript. Please update the draft accordingly.

**Response:** Thank you for your suggestions. To address your comment, I included a section in the Introduction on the importance of biosecurity measures: “The circulation of HPAI viruses in wild waterfowl populations has reinforced the importance of farm biosecurity as a measure for preventing virus introduction into poultry flocks. Because wild waterfowl detections may precede outbreaks in commercial poultry, surveillance of wild waterfowl can provide an early warning for implementing biosecurity measures. Consequently, periods of elevated HPAI activity in wild waterfowl should prompt enhanced biosecurity on poultry farms, including the exclusion of wild birds from poultry houses, feed, and water sources; the restriction of farm access; the use of dedicated clothing and footwear; and the routine cleaning and disinfection of vehicles, equipment, and personnel”

Additionally, I included a section in the Discussion section: “The observation that HPAI detections in wild waterfowl preceded poultry outbreaks suggests that wild bird surveillance can provide a valuable early warning period for implementing enhanced biosecurity measures. During periods of increased HPAI activity in wild birds, poultry producers should reinforce measures such as excluding wild birds from poultry facilities, protecting feed and water sources, restricting farm access, using dedicated clothing and footwear, and ensuring effective cleaning and disinfection of vehicles and equipment. Such targeted interventions may reduce the risk of virus introduction at the wild waterfowl-poultry interface.”

• Line 39-47: In the introduction, it is essential to include insights on the avian influenza virus to enhance readability and foster a comprehensive understanding of the topic. Furthermore, elaborating on genetic drift and shift is crucial, as these significant phenomena in influenza infections can critically influence the potential emergence of new epidemics or pandemics, underscoring the importance of their thorough explanation.

**Response:** The Introduction section was updated and includes the following: “ Avian influenza can undergo genetic drift and shift, forming new viral strains, and allowing it to adapt to new host species.”

“The current HPAI H5 strains show heterogeneity in vector competence and migratory behavior, are stable in the environment, have expanded their host tropism, including spillover into mammals, and can cause die-offs in waterfowl”.

• In the materials and methods section, consider mentioning the impact of H5 on humans. Although not significant, there have been a few reported cases. The CDC site link is provided for reference. The author can include this information here, in the discussion, or elsewhere as they see fit. This will expand the scope of the article.
<https://www.cdc.gov/bird-flu/situation-summary/index.html>

**Response:** We included in the Introduction section the following: “Although the risk to the public remains low, sporadic human H5N1 infections have been reported in individuals with direct exposure to infected animals, underscoring the zoonotic potential of these viruses and the need for integrated surveillance across wildlife, livestock, and human populations “

• It is crucial to clearly identify the mode of transmission among birds. Updating this information in the introduction or discussion section is essential to enhance the clarity and impact of the message.

**Response:** We included the following in the Introduction section:

“Transmission of HPAI H5 from wild birds to poultry can occur through both direct contact and indirect pathways, including contact with infected waterfowl, contaminated water sources, fomites, equipment, vehicles, and personnel. These transmission routes play a role in virus introduction into commercial poultry operations and viral transmission within and between farms

• The conclusion must be updated, as its current form does not accurately reflect the study's findings and is inconsistent with the abstract. An effective conclusion is essential to clearly convey the significance of the research. Please revise accordingly.

**Response:** Thank you for your comment. We updated the Conclusion section and included the following:

“This study identified distinct spatiotemporal patterns of HPAI H5 detections in major wild waterfowl species and HPAI H5 outbreaks in commercial poultry sectors across the United States between 2022 and 2025. The observed clustering patterns supported our hypothesis that wild waterfowl detections and poultry outbreaks would exhibit significant space-time clustering and spatial-temporal correspondence within major migratory flyways. Moreover, these associations varied among waterfowl species and poultry production sectors. Several putative spillover events were inferred from significant spatiotemporal associations between wild waterfowl and poultry clusters. The recurring observation that wild waterfowl clusters preceded or overlapped poultry outbreak clusters highlights the potential value of integrated wild bird surveillance as an early warning component of poultry health management. Collectively, these findings support risk-based surveillance and enhanced biosecurity measures tailored to local wild bird populations, migratory flyways, and poultry production systems.”

**Reviewer #2:**

This study addresses an important and timely topic in veterinary epidemiology and disease ecology by investigating the spatiotemporal relationship between HPAI H5 detections in wild waterfowl and outbreaks in commercial poultry across the United States. The integration of wildlife surveillance and poultry outbreak datasets represents a valuable One Health perspective, and the use of space-time scan statistics provides a useful descriptive framework for identifying potential hotspots of transmission risk. The findings regarding species-specific clustering patterns and differential associations between waterfowl species and poultry sectors may contribute to the improvement of risk-based surveillance and biosecurity programs. However, several methodological and interpretative concerns limit the strength of the causal inferences and epidemiological conclusions. In particular, the study overstates spatiotemporal co-occurrence as evidence of "spillover" and "transmission pathways" without sufficient evidence.

**Response:** Thank you for your comments. I agree that with the type of analyses we cannot infer causation. We updated the manuscript to reflect that.

Major Concerns
1. The study overinterprets spatiotemporal overlap between wild bird clusters and poultry outbreaks as direct evidence of spillover events. The space-time permutation model is appropriate when the population at risk are unavailable, but the resulting expected cases reflect the assumption of spatial-temporal independence rather than underlying population risk. Consequently, the reported O/E values quantify excess clustering relative to the null distribution in the space-time permutation model, but should not be interpreted as measures of infection risk or transmission intensity. Examples include statements such as: "Mallard detections preceded outbreaks in Northeast egg-layer and duck farms". The virus sequencing, phylogenetic analyses, and transmission modeling are needed to infer actual spillover events and pathways.

**Response:** Thank you for your comment. We agree that without sequencing of the virus, we cannot prove causation. To address your concern, we have revised the manuscript throughout to avoid causal language and to distinguish epidemiological associations from confirmed transmission events. Specifically, we now refer to these occurrences as putative spillover events inferred from significant spatiotemporal associations between wild bird detections and poultry outbreaks. We have also added a new table summarizing the wild bird clusters, poultry clusters, temporal relationships, geographic proximity, and supporting statistical evidence for each event.

In addition, we have expanded the Discussion and Limitations sections to emphasize that confirmation of transmission pathways would require complementary evidence, including viral genomic sequencing, phylogenetic analyses, and transmission modeling. Therefore, the findings should be interpreted as evidence of epidemiologically plausible links between wild bird and poultry outbreaks rather than confirmation of direct spillover.

2. The observed spatiotemporal clusters may be influenced by variable surveillance intensity. Wild bird surveillance is highly variable across states, species, seasons, and hunting activities; therefore, the observed distribution of detected HPAI may simply reflect differences in surveillance intensity and sampling effort rather than true in epidemiological differences. Areas with more intensive surveillance are inherently more likely to generate detections and subsequently be identified as significant clusters. A detailed discussion about the surveillance bias should be included as one of the limitations.

**Response:** Thank you for this important comment. We agree that variation in surveillance intensity represents a potential source of bias in analyses based on passive and active wildlife surveillance data. Detection probabilities can differ among states, species, seasons, hunter-harvest activities, and reporting effort, potentially influencing the spatial distribution of reported HPAI detections. However, it is important to note that the U.S. HPAI surveillance system is a large-scale, coordinated national program involving federal and state agencies and is specifically designed to monitor avian influenza across major migratory flyways and wild bird species.

To address the surveillance bias issue, we included the following:

“In addition, HPAI surveillance in the U.S. is conducted through a comprehensive national monitoring system targeting multiple wild bird species and migratory flyways; however, variation in surveillance intensity among regions, species, and seasons may have influenced detection probabilities and consequently the identification of some space-time clusters.”

3. The identification of the seven proposed spillover events rely on temporal and spatial overlap between wild bird and poultry clusters. However, no viral sequence data, statistical analyses, or other relevant information are provided to demonstrate these clusters as distinct spillover events. The authors should consider presenting these events in a table summarizing the wild bird clusters, poultry clusters, spatial proximity, temporal relationships, and supporting statistical evidence.

**Response:** Thank you for your suggestion. I agree that spatial-temporal overlap alone cannot definitively demonstrate direct spillover between wild waterfowl and poultry. To address this concern, we have revised the manuscript by adding a new table, Table 3, summarizing the proposed putative spillover events, including the associated wild waterfowl and poultry space-time clusters, geographic region, temporal relationships, and statistical evidence.

Specifically, the new table presents: (i) the wild waterfowl species and cluster identifiers, (ii) the corresponding poultry sector clusters, (iii) the time periods of both wild bird detections and poultry outbreaks, (iv) the temporal overlap or succession between clusters, (v) the geographic region/flyway where the clusters occurred, and (vi) the scan statistic and associated p-values for each cluster. This provides a transparent summary of the epidemiological evidence supporting each proposed event.

We have also revised the text throughout the manuscript to refer to these occurrences as "putative spillover events" rather than confirmed spillover events. We now acknowledge that our inferences are based on statistically significant spatiotemporal associations identified through retrospective space-time scan statistics and that confirmation of transmission pathways would require additional evidence, such as viral genomic sequencing and phylogenetic analyses.

We included the following as one of the limitations of our study: ”The absence of viral genomic and phylogenetic data precluded direct confirmation of wild bird-to-poultry transmission pathways.”

Minor Concerns
1. The rationale for limiting the analysis to Mallard, Canada Goose, Snow Goose, and Green-winged Teal requires further clarification. The study does not establish that they are the most epidemiologically important species at the wild bird-poultry interaction level. Excluding other potentially relevant waterfowl species may introduce a selection bias and could influence the interpretation of species-specific transmission patterns.

**Response:** Thank you for your comment. We agree that other waterfowl species may contribute to HPAI ecology and that no single set of species can fully represent the diversity of wild bird hosts involved in virus maintenance and spread. My intent was not to imply that Mallard, Canada Goose, Snow Goose, and Green-winged Teal are the only or necessarily the most epidemiologically important species involved in wild bird-to-poultry transmission. These four species were selected because they represented the most frequently detected wild waterfowl species in the dataset, collectively accounting for 39.7% of all laboratory-confirmed detections during the study period. In addition, they exhibited broad geographic distributions across multiple migratory flyways and had enough detections to support robust space-time cluster analyses. Species with substantially fewer detections often produced sparse spatial distributions and insufficient case counts for reliable cluster identification, thereby limiting meaningful species-specific spatiotemporal analyses.

To clarify this rationale, we have revised the Methods section to emphasize that species selection was based on a combination of detection frequency, geographic coverage, and adequate sample size for statistical analysis, rather than an assumption that these species are uniquely responsible for poultry introductions. We also acknowledge this as a limitation of the study and note that future investigations incorporating additional waterfowl species may provide an understanding of species-specific transmission dynamics.

2. Commercial poultry outbreaks were analyzed as counts of outbreak events rather than incidence rates. Regions with high poultry production density may naturally experience a greater number of outbreaks, and the detected clusters may only partially reflect the locations of the poultry operations rather than the transmission risk.

**Response:** Thank you for your observation. I agree that areas with greater poultry production density may experience a larger number of outbreaks, and therefore clusters identified using outbreak counts could partially reflect the underlying spatial distribution of poultry operations. Ideally, the analysis would incorporate denominators such as the number of poultry premises or poultry population at risk to estimate incidence rates and better distinguish outbreak risk from production density. However, comprehensive and temporally determined national data on poultry premises and bird inventories were not available for all poultry sectors included in this study. Consequently, our objective was to identify significant concentrations of reported outbreaks in space and time rather than estimate incidence-based risk. The retrospective space-time permutation scan statistic was selected because it is designed for analyses in which population-at-risk denominators are unavailable, evaluating whether observed cases are more concentrated in space and time than expected based on the distribution of all reported cases.

We have added this clarification to the Methods and Discussion sections and acknowledge that some detected poultry clusters may reflect regions with high poultry production density in addition to areas experiencing elevated transmission. Future studies incorporating poultry population or premises-level denominator data would allow incidence-based analyses and provide a direct assessment of outbreak risk.
