# Supplementary material for: Spatiotemporal clustering of highly pathogenic avian influenza (HPAI) H5N1 at the wild waterfowl-poultry interface: Vector-specific spillover risks in the U.S., 2022–2025
Source: PLoS One. 2026 Sep 23;21(9):e0345354. doi: 10.1371/journal.pone.0345354 (PMC13600479; doi:10.1371/journal.pone.0345354)
Supplement: Peer review history

# Peer review: Decision Letter 0

**DOI:** 10.1371/journal.pone.0345354.r001

**Authors:** Nickson Otieno

11 Jun 2026

Dear Dr. Varga,

Thank you for submitting your manuscript to PLOS ONE. After careful consideration, we feel that it has merit but does not fully meet PLOS ONE’s publication criteria as it currently stands. Therefore, we invite you to submit a revised version of the manuscript that addresses the points raised during the review process.

The reviewers cited several shortcomings that make the article unsuitable for acceptance to publish in the present form. These short are widespread across the various sections of the paper but mostly in the methodological protocol, and as a result of which the results presented and discussed, and from which conclusions are drawn are largely in discordance.

In particular:

- Temporal and spatial variations on bird/poultry observations is interpreted to imply infection risk spillover, without demonstrated epidemiological evidence through analyses of viral sequencing
- Potential biases in regional (state to state) levels of variation in poultry production intensity or surveillance in relation to risk infection, is not accounted for in the analyses
- Need for more clearer and transparent presentation of results in tabular form an in a more complete/comprehensive way
- The choice of the range of study species of wild birds is not justified in the Methods (i.e. why these specie in particular yet they are not necessarily the most vulnerable, or the most significant risk carriers?)

Many more specific issues are highlighted by the reviewers and need to be addressed in point by point form in a separate document to accompany the major revision of the paper

Two other major outstanding drawbacks of the paper are:

1. The very small number of literature cited literature (24). For a subject with such a large proliferation of research in the last 5 years, this small number of citations clearly indicates serious in adequacy in literature review and subliminal justification f the gap that the study serves to fill. It might actually turn out that this topic has already been researched and published in recent times, and so it is necessary to conduct a thorough literature search (at least 60 % no older than 10 years) in the Introduction section to more clearly demonstrate novelty for your particular topic. The introduction needs a lot more expansion to provide a broader background information to the study. AS one reviewer has suggested, elements such as genetic drifts and the transmission mode for the virus, must be mentioned as early as possible
2. At the end of the Introduction section, there is no clear indication of what hypotheses were tested to justify the modeling, or at least a statement of what the study expected to find. This is necessary and should be clearly to bot the knowledge gap addressed and the specific objectives pursued, as well as reflected in the Conclusion, to show whether those hypotheses were ultimately proved or not

For the revised paper, the authors must also adhere more closely to the journal’s forming rules according the Submission Guidelines, including font type and sizes, grammar and figure quality as well as sequential arrangement of the various  sections and numbering of in-text literature citations.

Please submit your revised manuscript by Jul 26 2026 11:59PM. If you will need more time than this to complete your revisions, please reply to this message or contact the journal office at plosone@plos.org. When you're ready to submit your revision, log on to https://www.editorialmanager.com/pone/ and select the 'Submissions Needing Revision' folder to locate your manuscript file.

- A letter that responds to each point raised by the academic editor and reviewer(s). You should upload this letter as a separate file labeled 'Response to Reviewers'.
- A marked-up copy of your manuscript that highlights changes made to the original version. You should upload this as a separate file labeled 'Revised Manuscript with Track Changes'.
- An unmarked version of your revised paper without tracked changes. You should upload this as a separate file labeled 'Manuscript'.

If you would like to make changes to your financial disclosure, please include your updated statement in your cover letter. Guidelines for resubmitting your figure files are available below the reviewer comments at the end of this letter.

If applicable, we recommend that you deposit your laboratory protocols in protocols.io to enhance the reproducibility of your results. Protocols.io assigns your protocol its own identifier (DOI) so that it can be cited independently in the future. For instructions see: https://journals.plos.org/plosone/s/submission-guidelines#loc-laboratory-protocols. Additionally, PLOS ONE offers an option for publishing peer-reviewed Lab Protocol articles, which describe protocols hosted on protocols.io. Read more information on sharing protocols at https://plos.org/protocols?utm_medium=editorial-email&utm_source=authorletters&utm_campaign=protocols.

As the corresponding author, your ORCID iD is verified in the submission system and will appear in the published article. PLOS supports the use of ORCID, and we encourage all coauthors to register for an ORCID iD and use it as well. Please encourage your coauthors to verify their ORCID iD within the submission system before final acceptance, as unverified ORCID iDs will not appear in the published article. *Only*  the individual author can complete the verification step; PLOS staff *cannot*  verify ORCID iDs on behalf of authors.

We look forward to receiving your revised manuscript.

Kind regards,

Nickson Erick Otieno, PhD

Academic Editor

PLOS One

Journal Requirements:

When submitting your revision, we need you to address these additional requirements.

1. Please ensure that your manuscript meets PLOS ONE's style requirements, including those for file naming. The PLOS ONE style templates can be found at

https://journals.plos.org/plosone/s/file?id=wjVg/PLOSOne_formatting_sample_main_body.pdf and

https://journals.plos.org/plosone/s/file?id=ba62/PLOSOne_formatting_sample_title_authors_affiliations.pdf

2. We note that All Figures in your submission contain map/satellite images which may be copyrighted. All PLOS content is published under the Creative Commons Attribution License (CC BY 4.0), which means that the manuscript, images, and Supporting Information files will be freely available online, and any third party is permitted to access, download, copy, distribute, and use these materials in any way, even commercially, with proper attribution. For these reasons, we cannot publish previously copyrighted maps or satellite images created using proprietary data, such as Google software (Google Maps, Street View, and Earth). For more information, see our copyright guidelines: http://journals.plos.org/plosone/s/licenses-and-copyright.

We require you to either (1) present written permission from the copyright holder to publish these figures specifically under the CC BY 4.0 license, or (2) remove the figures from your submission:

1. You may seek permission from the original copyright holder of All Figures to publish the content specifically under the CC BY 4.0 license.

We recommend that you contact the original copyright holder with the Content Permission Form (http://journals.plos.org/plosone/s/file?id=7c09/content-permission-form.pdf) and the following text:

“I request permission for the open-access journal PLOS ONE to publish XXX under the Creative Commons Attribution License (CCAL) CC BY 4.0 (http://creativecommons.org/licenses/by/4.0/). Please be aware that this license allows unrestricted use and distribution, even commercially, by third parties. Please reply and provide explicit written permission to publish XXX under a CC BY license and complete the attached form.”

Please upload the completed Content Permission Form or other proof of granted permissions as an ""Other"" file with your submission.

In the figure caption of the copyrighted figure, please include the following text: “Reprinted from [ref] under a CC BY license, with permission from [name of publisher], original copyright [original copyright year].”

2. If you are unable to obtain permission from the original copyright holder to publish these figures under the CC BY 4.0 license or if the copyright holder’s requirements are incompatible with the CC BY 4.0 license, please either i) remove the figure or ii) supply a replacement figure that complies with the CC BY 4.0 license. Please check copyright information on all replacement figures and update the figure caption with source information. If applicable, please specify in the figure caption text when a figure is similar but not identical to the original image and is therefore for illustrative purposes only.

The following resources for replacing copyrighted map figures may be helpful:

USGS National Map Viewer (public domain): http://viewer.nationalmap.gov/viewer/

The Gateway to Astronaut Photography of Earth (public domain): http://eol.jsc.nasa.gov/sseop/clickmap/

Maps at the CIA (public domain): https://www.cia.gov/library/publications/the-world-factbook/index.html and https://www.cia.gov/library/publications/cia-maps-publications/index.html

NASA Earth Observatory (public domain): http://earthobservatory.nasa.gov/

Landsat: http://landsat.visibleearth.nasa.gov/

USGS EROS (Earth Resources Observatory and Science (EROS) Center) (public domain): http://eros.usgs.gov/#

Natural Earth (public domain): http://www.naturalearthdata.com/

3. If the reviewer comments include a recommendation to cite specific previously published works, please review and evaluate these publications to determine whether they are relevant and should be cited. There is no requirement to cite these works unless the editor has indicated otherwise.

[Note: HTML markup is below. Please do not edit.]

Reviewers' comments:

Reviewer's Responses to Questions

**Comments to the Author**

1. Is the manuscript technically sound, and do the data support the conclusions?

Reviewer #1: Yes

Reviewer #2: Yes

**********

2. Has the statistical analysis been performed appropriately and rigorously?

Reviewer #1: Yes

Reviewer #2: No

**********

3. Have the authors made all data underlying the findings in their manuscript fully available?

The PLOS Data policy

Reviewer #1: Yes

Reviewer #2: No

**********

4. Is the manuscript presented in an intelligible fashion and written in standard English?

Reviewer #1: Yes

Reviewer #2: Yes

**********

Reviewer #1: Varga C. investigated spatiotemporal clustering of HPAI (H5N1) at the wild waterfowl-poultry interface in the USA and considered farm biosafety strategies to contain these recurrent, vector-specific risks. The study offers a thorough analysis, making this article a good read for understanding these spillovers.

Below are the comments-

• Overall, this is a well-written research article on a worthy topic supported by good methodologies and results. Further improvements and revisions will undoubtedly enhance its overall quality.

• The title reflects the study well.

• The abstract aligns well with the study and conveys it effectively. Further updates in the abstract will definitely enhance its overall quality.

Comments:

• Line 34: In the conclusion section of the abstract, the author highlighted the critical importance of farm biosecurity strategies, which must be given more emphasis in the manuscript. Please update the draft accordingly.

• Line 39-47: In the introduction, it is essential to include insights on the avian influenza virus to enhance readability and foster a comprehensive understanding of the topic. Furthermore, elaborating on genetic drift and shift is crucial, as these significant phenomena in influenza infections can critically influence the potential emergence of new epidemics or pandemics, underscoring the importance of their thorough explanation.

• In the materials and methods section, consider mentioning the impact of H5 on humans. Although not significant, there have been a few reported cases. The CDC site link is provided for reference. The author can include this information here, in the discussion, or elsewhere as they see fit. This will expand the scope of the article.

https://www.cdc.gov/bird-flu/situation-summary/index.html

• It is crucial to clearly identify the mode of transmission among birds. Updating this information in the introduction or discussion section is essential to enhance the clarity and impact of the message.

• The conclusion must be updated, as its current form does not accurately reflect the study's findings and is inconsistent with the abstract. An effective conclusion is essential to clearly convey the significance of the research. Please revise accordingly.

Reviewer #2: This study addresses an important and timely topic in veterinary epidemiology and disease ecology by investigating the spatiotemporal relationship between HPAI H5 detections in wild waterfowl and outbreaks in commercial poultry across the United States. The integration of wildlife surveillance and poultry outbreak datasets represents a valuable One Health perspective, and the use of space-time scan statistics provides a useful descriptive framework for identifying potential hotspots of transmission risk. The findings regarding species-specific clustering patterns and differential associations between waterfowl species and poultry sectors may contribute to the improvement of risk-based surveillance and biosecurity programs. However, several methodological and interpretative concerns limit the strength of the causal inferences and epidemiological conclusions. In particular, the study overstates spatiotemporal co-occurrence as evidence of "spillover" and "transmission pathways" without sufficient evidence.

Major Concerns

1. The study overinterprets spatiotemporal overlap between wild bird clusters and poultry outbreaks as direct evidence of spillover events. The space-time permutation model is appropriate when the population at risk are unavailable, but the resulting expected cases reflect the assumption of spatial-temporal independence rather than underlying population risk. Consequently, the reported O/E values quantify excess clustering relative to the null distribution in space-time permutation model, but should not be interpreted as measures of infection risk or transmission intensity. Examples include statements such as: "Mallard detections preceded outbreaks in Northeast egg-layer and duck farms". The virus sequencing, phylogenetic analyses, and transmission modeling are needed to infer actual spillover events and pathways.

2. The observed spatiotemporal clusters may be influenced by variable surveillance intensity. Wild bird surveillance is highly variable across states, species, seasons, and hunting activities; therefore, the observed distribution of detected HPAI may simply reflect differences in surveillance intensity and sampling effort rather than true in epidemiological differences. Areas with more intensive surveillance are inherently more likely to generate detections and subsequently be identified as significant clusters. A detailed discussion about the surveillance bias should be included as one of the limitations.

3. The identification of the seven proposed spillover events rely on temporal and spatial overlap between wild bird and poultry clusters. However, no viral sequence data, statistical analyses, or other relevant information are provided to demonstrate these clusters as distinct spillover events. The authors should consider presenting these events in a table summarizing the wild bird clusters, poultry clusters, spatial proximity, temporal relationships, and supporting statistical evidence.

Minor Concerns

1. The rationale for limiting the analysis to Mallard, Canada Goose, Snow Goose, and Green-winged Teal requires further clarification. The study does not establish that they are the most epidemiologically important species at the wild bird-poultry interaction level. Excluding other potentially relevant waterfowl species may introduce a selection bias and could influence the interpretation of species-specific transmission patterns.

2. Commercial poultry outbreaks were analyzed as counts of outbreak events rather than incidence rates. Regions with high poultry production density may naturally experience a greater number of outbreaks, and the detected clusters may only partially reflect the locations of the poultry operations rather than the transmission risk.

**********

what does this mean?). If published, this will include your full peer review and any attached files.

If you choose “no”, your identity will remain anonymous but your review may still be made public.

**Do you want your identity to be public for this peer review?**  For information about this choice, including consent withdrawal, please see our Privacy Policy

Reviewer #1: No

Reviewer #2: No

**********

[NOTE: If reviewer comments were submitted as an attachment file, they will be attached to this email and accessible via the submission site. Please log into your account, locate the manuscript record, and check for the action link "View Attachments". If this link does not appear, there are no attachment files.]

To ensure your figures meet our technical requirements, please review our figure guidelines: https://journals.plos.org/plosone/s/figures

You may also use PLOS’s free figure tool, NAAS, to help you prepare publication quality figures: https://journals.plos.org/plosone/s/figures#loc-tools-for-figure-preparation.

NAAS will assess whether your figures meet our technical requirements by comparing each figure against our figure specifications.

# Author comment: Author response to Decision Letter 1

**DOI:** 10.1371/journal.pone.0345354.r002

10 Aug 2026

The Response to reviewers comments was uploaded as a separate Word document file.

Attachment Submitted filename: Response to Reviewers.docx

# Peer review: Decision Letter 1

**DOI:** 10.1371/journal.pone.0345354.r003

**Authors:** Nickson Otieno

19 Aug 2026

Spatiotemporal clustering of highly pathogenic avian influenza (HPAI) H5N1 at the wild waterfowl-poultry interface: Vector-specific spillover risks in the U.S., 2022–2025

PONE-D-26-10950R1

Dear Dr. Varga,

We’re pleased to inform you that your manuscript has been judged scientifically suitable for publication and will be formally accepted for publication once it meets all outstanding technical requirements.

Within one week, you’ll receive an e-mail detailing the required amendments. When these have been addressed, you’ll receive a formal acceptance letter and your manuscript will be scheduled for publication.

An invoice will be generated when your article is formally accepted. Please note, if your institution has a publishing partnership with PLOS and your article meets the relevant criteria, all or part of your publication costs will be covered. Please make sure your user information is up-to-date by logging into Editorial Manager at Editorial Manager® and clicking the ‘Update My Information' link at the top of the page. For questions related to billing, please contact billing support.

If your institution or institutions have a press office, please notify them about your upcoming paper to help maximize its impact. If they’ll be preparing press materials, please inform our press team as soon as possible -- no later than 48 hours after receiving the formal acceptance. Your manuscript will remain under strict press embargo until 2 pm Eastern Time on the date of publication. For more information, please contact onepress@plos.org.

Kind regards,

Nickson Erick Otieno, PhD

Academic Editor

PLOS One

Additional Editor Comments (optional):

Reviewers' comments:

Reviewer's Responses to Questions

**Comments to the Author**

Reviewer #1: All comments have been addressed

**********

2. Is the manuscript technically sound, and do the data support the conclusions?

Reviewer #1: Yes

**********

3. Has the statistical analysis been performed appropriately and rigorously?

Reviewer #1: Yes

**********

4. Have the authors made all data underlying the findings in their manuscript fully available?

The PLOS Data policy

Reviewer #1: Yes

**********

5. Is the manuscript presented in an intelligible fashion and written in standard English?

Reviewer #1: Yes

**********

Reviewer #1: Thank you for addressing all comments and providing comprehensive responses. The revised manuscript demonstrates improved overall quality.

**********

what does this mean?). If published, this will include your full peer review and any attached files.

If you choose “no”, your identity will remain anonymous but your review may still be made public.

**Do you want your identity to be public for this peer review?**  For information about this choice, including consent withdrawal, please see our Privacy Policy

Reviewer #1: No

**********

# Editor report: Acceptance letter

**DOI:** 10.1371/journal.pone.0345354.r004

**Authors:** Nickson Otieno

PONE-D-26-10950R1

PLOS One

Dear Dr. Varga,

I'm pleased to inform you that your manuscript has been deemed suitable for publication in PLOS One. Congratulations! Your manuscript is now being handed over to our production team.

At this stage, our production department will prepare your paper for publication. This includes ensuring the following:

* All references, tables, and figures are properly cited

* All relevant supporting information is included in the manuscript submission,

* There are no issues that prevent the paper from being properly typeset

You will receive further instructions from the production team, including instructions on how to review your proof when it is ready. Please keep in mind that we are working through a large volume of accepted articles, so please give us a few days to review your paper and let you know the next and final steps.

Lastly, if your institution or institutions have a press office, please let them know about your upcoming paper now to help maximize its impact. If they'll be preparing press materials, please inform our press team within the next 48 hours. Your manuscript will remain under strict press embargo until 2 pm Eastern Time on the date of publication. For more information, please contact onepress@plos.org.

You will receive an invoice from PLOS for your publication fee after your manuscript has reached the completed accept phase. If you receive an email requesting payment before acceptance or for any other service, this may be a phishing scheme. Learn how to identify phishing emails and protect your accounts at https://explore.plos.org/phishing.

If we can help with anything else, please email us at customercare@plos.org.

Thank you for submitting your work to PLOS One and supporting open access.

Kind regards,

PLOS One Editorial Office Staff

on behalf of

Dr. Nickson Erick Otieno

Academic Editor

PLOS One
